# *LsNAC46* regulates heat-resistance and growth in lettuce (*Lactuca sativa* L.)

**DOI:** 10.3389/fpls.2025.1560470

**Published:** 2025-08-19

**Authors:** Tao Yang, Shu-ping He, Guo-tao Huo, Ming-lu Tian, Li Du, Xiao Yang, Heng Xu, Guo-jun Ge, Hong-yang Zhao, Li-jun Luo, Shi-wei Wei

**Affiliations:** ^1^ Shanghai Agrobiological Gene Center, Shanghai, China; ^2^ Institute of Agricultural Science and Technology Information, Shanghai Academy of Agricultural Sciences, Shanghai, China

**Keywords:** lettuce, heat resistant, NAC, overexpression, achene weight

## Abstract

Lettuce prefers a cold and cool climate, and high temperatures can lead to many problems such as tip burn that decrease yield and quality. NAC (NAM, ATAF1/2, and CUC2) proteins are important regulatory factors in abiotic stress responses. In our previous transcriptomic analysis, we identified that *LsNAC46* is involved in the response to heat stress in lettuce. This study reports that LsNAC46 (LOC111911762), a member of the NAC transcription factor family, has transcriptional activation activity, and regulates heat tolerance, growth, and development in lettuce. Tissue expression analysis showed the highest expression levels of *LsNAC46* in lettuce achene. Compared with wild-type (WT) plants, *LsNAC46-*overexpressing lines had a higher heat damage index and lower survival rate when exposed to high temperature, accompanied by reduced accumulation of total protein (TP), proline (PRO), and phenylalanine ammonia-lyase (PAL), as well as a lower Fv/Fm, indicating that *LsNAC46* negatively regulates heat tolerance in lettuce. In addition, the plant width, leaf length, and fresh weight were significantly lower, but the achene weight was significantly higher in *LsNAC46-*overexpressing plants. This study indicates that *LsNAC46* may regulate heat stress responses by regulating intracellular enzyme activity, osmotic pressure, photosynthetic capacity, and synthesis of secondary metabolites. This knowledge may enable the characterization of *LsNAC46* in the response to heat stress and regulation of plant stress responses or achene weight.

## 1 Introduction

Lettuce (*Lactuca sativa*.L), a member of the *Asteraceae* family, is a self-compatible, diploid vegetable with 18 chromosomes that is an important green leafy vegetable widely cultivated in the world. Domesticated lettuce is rich in various nutrients such as polyphenols, lactucin, vitamins, protein and sugars. Lettuce originated in a cool Mediterranean region, and is sensitive to high temperatures, which limits the growth and development of the plants.

In recent years, global warming has become an increasingly serious issue and is resulting in frequent heat stress to vegetable crops. Heat stress can cause a series of morphological and physiological changes that significantly affect the growth and yield of plants (Shah et al., 2011; Qu et al., 2013; Wang et al., 2024). In order to adapt to adverse environments, plants can respond to stress through physiological and biochemical changes and specific signaling pathways (Fang et al., 2015). Throughout evolution, plants have developed a complex antioxidant enzyme system to remove excess reactive oxidative species (ROS) (Choudhury et al., 2017). The antioxidant system can also enhance the adaptability of plants to heat stress by increasing the content of osmotic regulatory substances (Hare et al., 2010), such as proline (PRO), soluble sugars (SS), and soluble proteins (SP). Plants adapt to abiotic stresses through a complex regulatory process that involves physiological indicators, such as TP, PRO, and PAL. For example, under salt stress conditions, overexpression of *PvLBD12* can induce the accumulation of proline and thereby enhance the salt tolerance of transgenic switchgrass (*Panicum virgatum* L.) (Guan et al., 2023).

NAC transcription factors are one of the largest gene families in plants. In recent years, NAC (NAM, ATAF1/2, and CUC2) transcription factors have been demonstrated to function as key regulatory factors that respond to abiotic stress (Swati et al., 2012). Numerous whole genome analyses of the NAC family have been conducted, such as the discovery of 101, 152, 151, and 117 NAC family members in soybean, corn, rice, and *Arabidopsis*, respectively (Mohammed et al., 2010; Le et al., 2011; Shang et al., 2013; Kaliyugam et al., 2014). In lettuce, there are a total of 99 members of the NAC genes family (Du et al., 2021). NAC transcription factors are typically composed of approximately 150–160 amino acids and contain a highly conserved protein domain at their N-terminus that consists of five sub domains: A, B, C, D, and E. The C-terminus is a highly variable transcriptional regulatory region involved in transcriptional activation or inhibition (Mohanta et al., 2020).

NAC genes have been reported to play important roles in plant stress resistance, growth and development, secondary metabolite synthesis, and defense against pathogens (Swati et al., 2012). There have been many studies on the involvement of NAC genes and other transcription factors in plant responses to high-temperature stress in plant species such as rice, tomato, and *Arabidopsis*. The *RD26* gene was the first NAC gene identified to participate in abscisic acid (ABA) and jasmonate (JA) signaling during the stress period in *Arabidopsis* (Fujita et al., 2004; Yamaguchi and Shinozaki, 2007). By screening 152 recombinant inbred lettuce lines, Jenni identified a major quantitative trait locus (QTL) (*qTPB5.2*) as a good molecular marker for a future leaf tip-burning phenotype (Jenni et al., 2013). In *Arabidopsis*, overexpression of *ANAC042* significantly increased the survival rate of *Arabidopsis* under heat stress (Shahnejat-Bushehri et al., 2012). Moreover, overexpression of *ANAC019* enhanced the heat tolerance of transgenic *Arabidopsis* plants by inducing the expression of a series of heat shock proteins (Guan et al., 2014). Overexpression of *TaNAC2L* significantly improved the high-temperature tolerance of *Arabidopsis* by upregulating other stress-related proteins (RD17, LEA, HSP70, RD29A) (Guo et al., 2015). In rice, overexpression of *OsNAC5* significantly improved stress resistance and overexpression of *OsNAC3* significantly improved heat tolerance (Sperotto et al., 2009; Jeong et al., 2010). However, *ATAF1* transgenic plants for thermomemory showed a strong phenotype, where the ataf1–2 and ataf1–4 mutants had a significantly higher survival rate and fresh mass compared with WT plants, while plants overexpressing *ATAF1* exhibited a severely reduced thermomemory (Alshareef et al., 2022). These studies are very compressive in rice and other crops, but much information is missing for lettuce.

Although the functions of NAC genes have been reported in other plant species, our study represents the first report on NAC genes functionality in lettuce (*Lactuca sativa* L.). In our previous study, we identified that *LsNAC46* contributes to under-heat stress responses in lettuce by transcriptomic analysis, *LsNAC46* was upregulated in lettuce seedlings exposed to heat stress, therefore, we selected this gene for further functional verification (Wei et al., 2021). In this study, we further investigated the function of *LsNAC46* in lettuce and reported that *LsNAC46* exerts transcriptional activation activity and is expressed at the highest levels in achene. Moreover, overexpressing *LsNAC46* significantly reduced plant width, leaf length, and fresh weight, but increased achene weight. Under heat stress, the accumulation of total protein (TP), proline (PRO), and phenylalanine ammonia-lyase (PAL) was significantly lower in plants overexpressing *LsNAC46*, which suggests that *LsNAC46* may confer tolerance to heat stress by reducing the accumulation of total protein (TP), proline (PRO) and phenylalanine ammonia-lyase (PAL) under heat stress, which decreased heat tolerance in lettuce. Overall, this research indicates that *LsNAC46* plays an important role in the response to heat stress and could be a useful and effective genetic target to develop lettuce with higher achene yield.

## 2 Materials and methods

### 2.1 Plant materials and stress treatment

Lettuce variety ‘WD40AA’ was collected from the Huazhong Agricultural University; ‘KNV482’ was obtained from the Shanghai Agrobiological Gene Center. Plants were subjected to heat stress treatment for 3 d and 5 d after growing under normal conditions for 25 d and 45 d. Normal growth conditions: the lettuce plants were grown in growth chambers, 16 h light at 24°C and 8 h dark at 20°C. Heat stress conditions: 16 h light at 38°C and 8 h dark at 33°C. The humidity of the growth environment was controlled at 60% and the daytime light intensity was maintained at 180-220 μmol • m^-2^ • s^-1^.

### 2.2 LsNAC46 protein structure and tissue expression analysis

Using the cDNA sequence of the gene sequence provided by NCBI as a template, we cloned full-length *LsNAC46* using a PCR cloning method. CDD (https://www.ncbi.nlm.nih.gov/Structure/cdd/wrpsb.cgi) was used to predict the conserved functional domains in the LsNAC46 protein. Protein Primary Structure Analysis Using ProtParam (http://web.expasy.org/protparam/) was conducted to predict and analyze the physicochemical properties regarding candidate NAC proteins. Protein secondary structure analysis was performed using the SOPMA program on the NPS server (https://npsa-prabi.ibcp.fr/cgibin/npsa_automat.pl?page=npsa_sopma.html). The upstream sequence of the start codon (ATG) of the *LsNAC46* gene was found in the lettuce gene pool, and the promoter region was analyzed and predicted using the PlantCARE promoter online tool.

‘WD40AA’ plants were grown until they bloomed (around 185 days). We collected new leaf, stem, old stem, flower, root, and achene at 3 days, 6 days, and 9 days after flowering, extracted total RNA, and measured the expression of *LsNAC46* in ‘WD40AA’ according to qRT-PCR.

### 2.3 Analysis of the promoter region and transcriptional activation activity

The upstream sequence of the *LsNAC46* gene was assessed using the PlantCARE promoter online analysis tool to predict promoter regions. Design primers with BD tags using plasmids containing target genes as templates for PCR amplification. After recovery and purification, the amplified product was recombined using ExnaseTM II enzyme, and the fragment of the recombinantion product was cloned into a yeast expression vector pGBKT7 to construct the pGBKT7-LsNAC46 expression vector. Monoclonal clones and PCR test-positive clones. Saccharomyces cerevisiae strain Y2H transformed with pGBKT7-LsNAC46 or empty vector (control) was serially diluted (10^0 to 10^-3) and plated on synthetic defined (SD) media: SD/-Trp (selection) and SD/-Trp-His (activation assay). Growth was monitored after 72 h incubation at 30°C.

### 2.4 Construction of overexpression vector and genetic transformation

To verify the function of the *LsNAC46* gene, a *LsNAC46* overexpression vector was constructed. The coding sequence of the target gene was amplified from ATG to the stop codon. Connector sequences were added to the 5’ end of the positive primer (CAAGTTCTACTGTTGATACATATAG) and the 5’ end of the reverse primer (CCCGGGGTCGACGGCATATAG). In our previous study, we identified *LsNAC46* contributes to under heat stress responses in lettuce ‘KNV482’ by transcriptomic analysis, PCR amplification was performed with cDNA as the template to obtain foreign fragments. The PRI101-GFP vector digested by NdeI was homologously recombined with the fragment and transformed into competent *E. coli* Trans-T1. We used Pri101-F/R primers to detect colonies containing the correct clone, obtain a positive monoclonal bacterial colony, and confirmed the plasmid by sequencing. The plasmids were transformed into sentient Agrobacterium GV3101 and the Pri101-F/R primers were used to detect *Agrobacterium* colonies expressing the plasmid. The full‐length cDNAs of *LsNAC46* was amplified and cloned into pRI101 vectors driven by *CaMV 35S* promoter. The procedure for the transformation of lettuce from the previously described method (Su et al., 2020). *Agrobacterium tumefaciens* strain GV3101 was transformed with the constructs that were used for plant transformation using the freeze-thaw method. Transgenic plants were generated using cotyledon explants. The genetic transformation receptor material is ‘WD40AA’ (WT). Transgenic plants were regenerated and identified by kanamycin resistance and PCR using a gene-specific primer (**Supplementary Table S2**). The homozygous progenies designated as OE-11, OE-18, and OE-20 represent three independent *LsNAC46* overexpression (OE) lines.

### 2.5 Determination of physiological indicators

The 25-day-old (WT, OE-11, OE-18 and OE-20 and WT) lettuce seedlings were treat by 3 d heat stress (16 h light/38°C, 8 h dark/33°C), with 3 replicates, 24 seedlings per replicates. Fv/Fm values were measured using a chlorophyll fluorescence imaging system after dark adaptation of the leaves for 20 minutes, strictly follow the operating procedures of the chlorophyll fluorescence imaging system (Model CF0069, Chlorophyll fluorescence Imager (CFI), UK Technologica). Survival rates were identified after 2-day recovery under normal conditions (16 h light/24°C, 8 h dark/20°C). Heat damage index scored following 3-day heat stress.


$$\text{ HI computing method}:\text{HI}=\frac{\Sigma (\text{Number of plants at each level }\times \text{Level)}}{\text{Maxinum }\times \text{Total number of plant}}\;\times 100$$

Classification criteria 0: lettuce leaves are normal with no heat damage; 1: 1–2 leaf tips and edges are burnt or rotten; 3: half of the leaves of lettuce plant are burnt or rotten; 5: two-thirds of the leaves of lettuce plant are burnt or rotten; 7: entire lettuce plant is burnt or rotten).

Lettuce plants were subjected to heat stress (16 h light at 38°C and 8 h dark at 33°C) for 5 d after growing under normal conditions for 45 d, with 3 replicates, 8 seedlings per replicates. The total protein content (TP), proline content (PRO) and phenylalanine ammonia-lyase activity (PAL) were measured by using leaf samples that harvested from the same part of the plant. The measured methods were used by reagent kits (Nanjing Jiancheng Biotechnology Research Institute) according to instructions of the physiological indicator kits. The leaf length, plant width and fresh weight were identified with five replicates (per replicate 12 seedlings). The seeds characters were measured with five replicates when all the WT and overexpression lines growth the same condition.

### 2.6 Statistical analysis

Data collation was performed using Microsoft Excel 2010, and statistical analyses were conducted using GraphPad Prism (v8.4.2.679). Student’s t-tests were utilized for comparisons between the two groups. One-way ANOVA was employed for comparisons among multiple groups. Graphs were generated using GraphPad Prism (v8.4.2.679).

## 3 Results

### 3.1 Analysis of LsNAC46 sequence and protein structure, promoter region analysis

**Figure 1:**
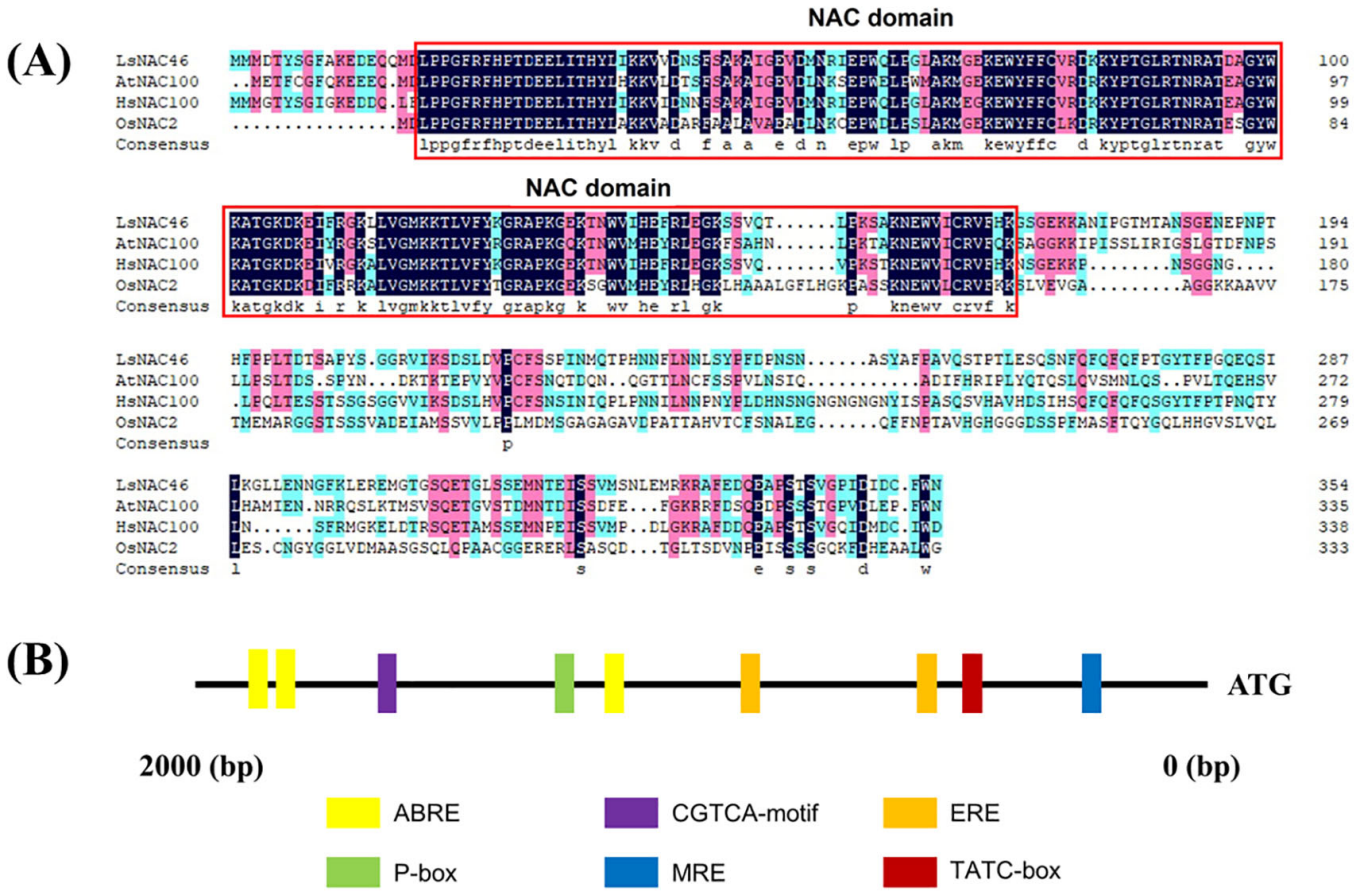
Multiple alignment of lettuce LsNAC46 and other species NACs proteins. **(A)** The NAC domains are highlighted by the red box. AtNAC100, HsNAC100, and OsNAC2 are homologous proteins of LsNAC46 in *Arabidopsis thaliana*, *Helianthus annuus*, and *Oryza sativa*, respectively. **(B)** The cis-regulatory element analysis of the LsNAC46 promoter region.

In our previous research, we identified six NAC transcription factors that were differentially expressed in lettuce after heat treatment, including LsNAC46 (Wei et al., 2021). LsNAC46 has 355 amino acids which is encoded by a gene with full-length coding sequence of 1608 bp locating on chromosome 1 in lettuce. The protein has a predicted molecular weight of 40068.16 Da, theoretical pI of 6.14, 43 negatively charged residues (Asp+Glu), 40 positively charged residues (Arg+Lys), an average hydrophilic coefficient of -0.650%, and an instability coefficient of 33.20. The average hydrophilic coefficient suggests that LsNAC46 is a hydrophilic protein, while the instability coefficient suggests the protein is stable. The predicted secondary structure of LsNAC46 protein is composed of 18.87% α-helices, 11.27% extended strands, 3.66% β-turns, and 66.20% random coils. AtNAC100, HsNAC100, and OsNAC2 are homologs of LsNAC46 in *Arabidopsis thaliana*, *Helianthus annuus*, and *Oryza sativa*, respectively. LsNAC46 has a NAC conserved domain in amino acids of 172–864 in its N-terminal region based on predictions by the Pfam website (http://pfam.xfam.org/), which suggests that LsNAC46 belongs to the NAC family. The NAC domains are highlighted by red box in (**Figure 1A**). Multiple sequence alignment of LsNAC46 with its orthologs AtNAC100 (Arabidopsis thaliana), HsNAC100 (Helianthus annuus), and OsNAC2 (Oryza sativa) revealed conserved NAC domain architecture (**Figure 1**). Structural annotation through the Pfam database (v37.3; http://pfam.xfam.org/) identified a canonical NAC domain (PF02365; E-value 4.9e-75) spanning residues 19–169 in the N-terminal region of LsNAC46, confirming its classification within the NAC transcription factor family. The conserved region showed 89% sequence identity with HsNAC100, 84% with AtNAC100, and 71% with OsNAC2, with divergence primarily occurring in loop regions between secondary structures. Subcellular localization prediction for LsNAC46 was performed using Plant-mPLoc (a multi-subcellular localization predictor available at http://www.csbio.sjtu.edu.cn/bioinf/plant-multi/), with bioinformatic analysis indicating nuclear localization.

Through promoter cis-element analysis, we identified multiple stress-responsive regulatory motifs in the NAC promoter region, notably including ABA-responsive elements (ABREs), ethylene-responsive factor binding sites (ERFs), P-box (gibberellin-responsive element), TATC-box(cis-acting element involved in gibberellin-responsiveness), CGTCA-motif(cis-acting regulatory element involved in the MeJA-responsiveness), and MYB transcription factor binding sites (MRE) (**Figure 1B**). The promoter region of the *LsNAC46* gene contains *cis*-acting elements related to stress and hormones (**Supplementary Table S1**).

### 3.2 LsNAC46 exerts transcriptional activation activity

**Figure 2:**
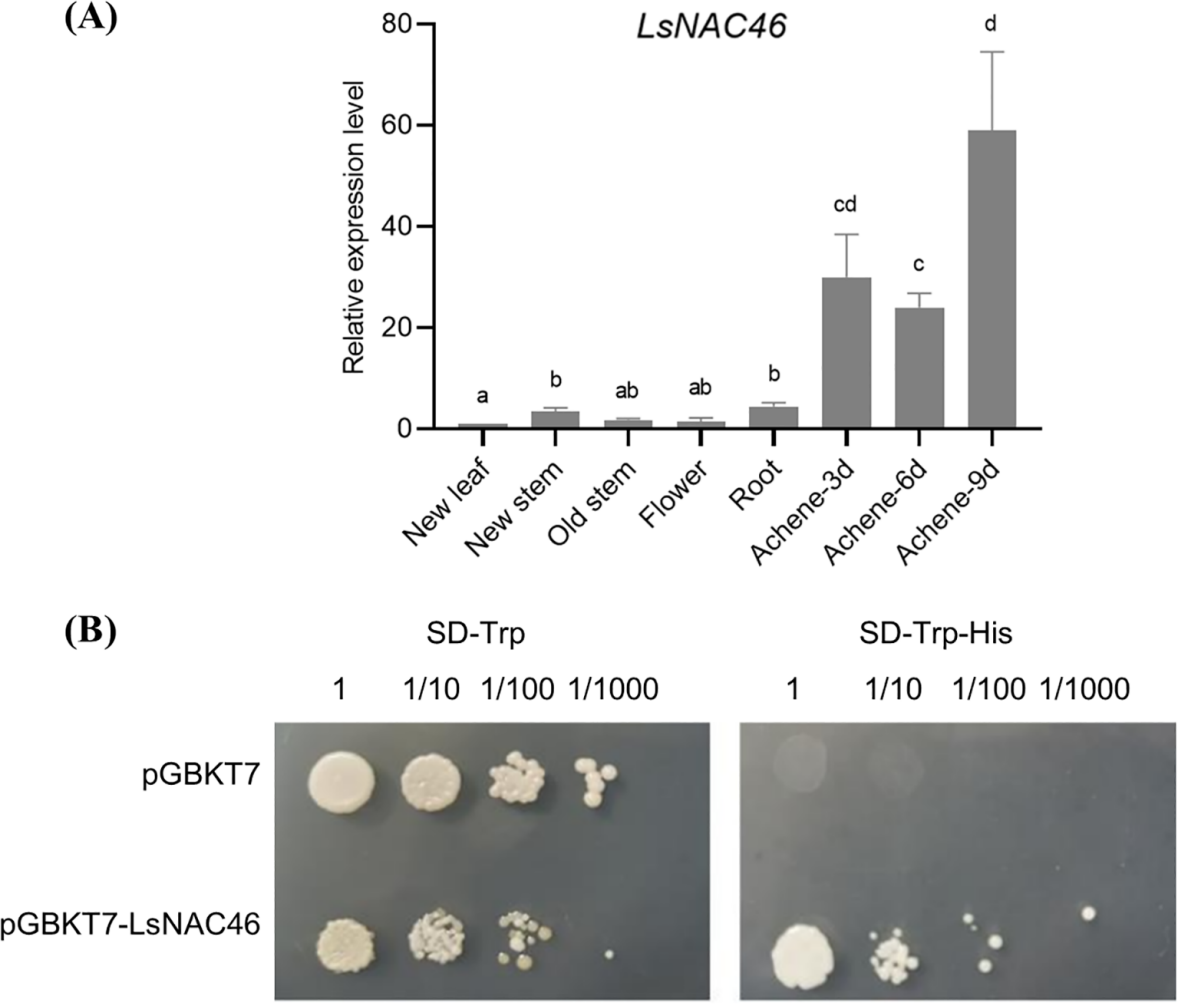
The expression of *LsNAC46* in different tissues and transcriptional activation of LsNAC46 in yeast. **(A)** Tissue-specific expression profiling of LsNAC46. Relative mRNA levels in roots, stems, mature leaves, and new leaves were quantified by qRT-PCR, with expression in new leaves normalized to 1.0. **(B)** Transcriptional activation assay in yeast. Saccharomyces cerevisiae strain Y2H transformed with pGBKT7-LsNAC46 or empty vector (control) was serially diluted (10^0 to 10^-3) and plated on synthetic defined (SD) media: SD/-Trp (selection) and SD/-Trp-His (activation assay). Growth was monitored after 72 h incubation at 30°C. Data represent mean ± SD of three biological replicates (n = 3 plants per line). Letters indicate significant differences between treatments based on Tukey’s test (P< 0.05).

The tissue expression (qRT-PCR) results showed that the relative expression level of *LsNAC46* was higher in the achene than in other tissues, with the highest expression level in 9-day-achene and the lowest expression level in flower and new leaf (**Figure 2A**). Transcriptional autoactivation assays showed that yeast containing the fusion expression vector pGBKT7-LsNAC46 can grow on SD/-Trp-His medium, which indicates that the LsNAC46 protein has self-activation activity (**Figure 2B**).

### 3.3 Confirmation of *LsNAC46*-overexpressing lines

**Figure 3:**
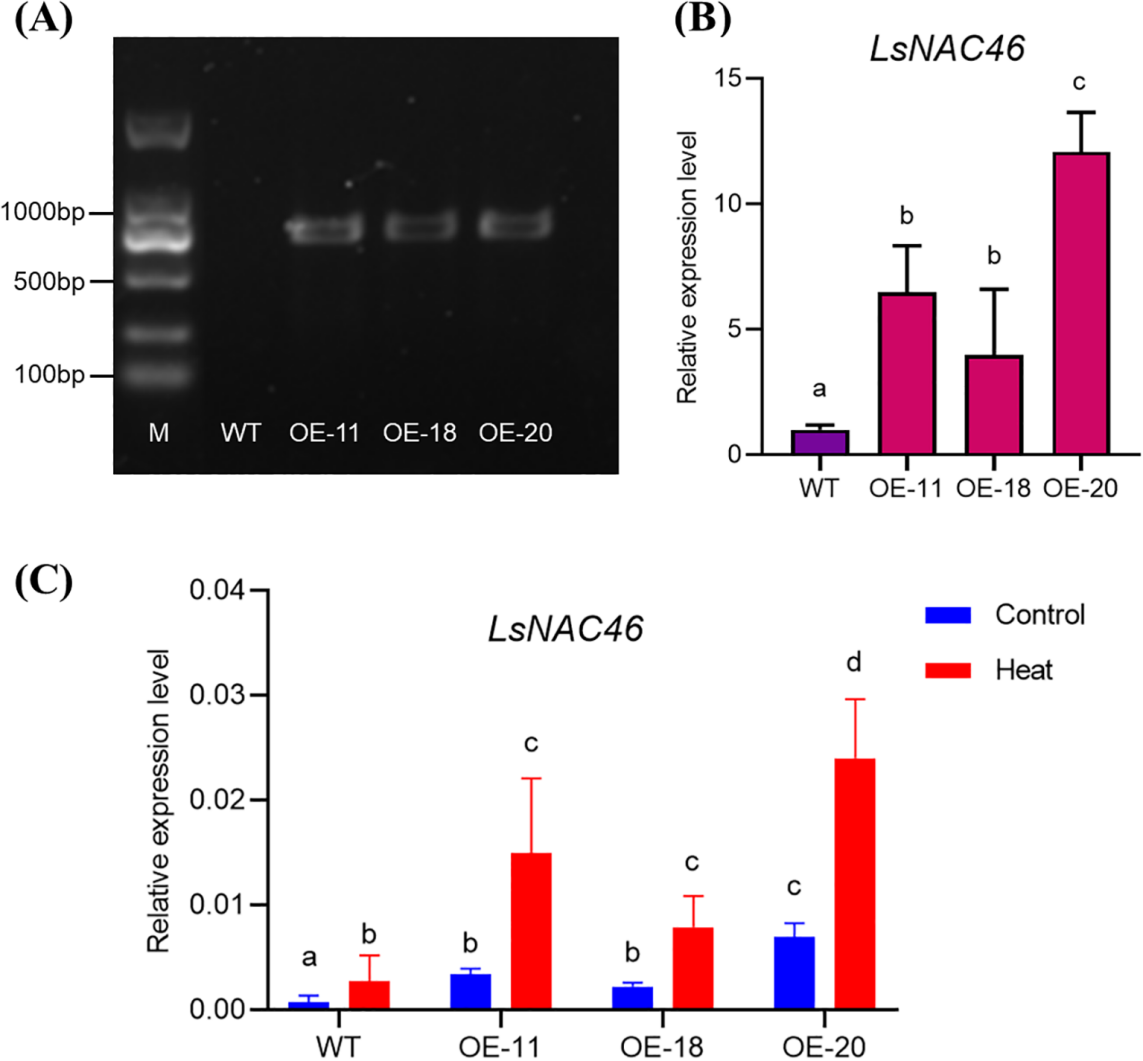
Validation of *LsNAC46*-overexpressing lettuce plants. **(A)** Molecular confirmation of transgenic lines by PCR. Lane M: DNA ladder; WT: non-transgenic control (‘WD40AA’ background). **(B)** Transcript abundance of *LsNAC46* in different transgenic lines. QRT-PCR analysis of *LsNAC46* expression in mature leaves of 25-day-old plants, normalized to WT levels (set as 1.0). **(C)** *LsNAC46* expression in WT and over expression lines under control and heat stress. Twenty-five-day-old lettuce plants were subjected to heat stress (16 h light/38°C, 8 h dark/33°C) for 72 h. Data represent mean ± SD of three biological replicates (n = 3 plants per line). Letters indicate significant differences between treatments based on Tukey’s test (P< 0.05).

To verify the function of *LsNAC46*, an overexpression vector was constructed and three overexpressing lines (OE-11, OE-18 and OE-20) were obtained by agrobacterium-mediated genetic transformation. Compared with the wild-type (WT: ‘WD40AA’), the OE-11, OE-18, and OE-20 lines led to amplification bands of around 1000 bp (**Figure 3A**) confirming insertion of the vector. The *LsNAC46*-overexpressing lines expressed higher levels of *LsNAC46* than WT; the OE-20 line had the highest expression level, 12.10 times higher than that of WT (**Figure 3B**). Expression of *LsNAC46* response to heat further confirmed that the *LsNAC46*-overexpressing plants were more expression levels (**Figure 3C**).

### 3.4 Overexpression of *LsNAC46* regulates the growth and development of lettuce plants and achene size

**Figure 4:**
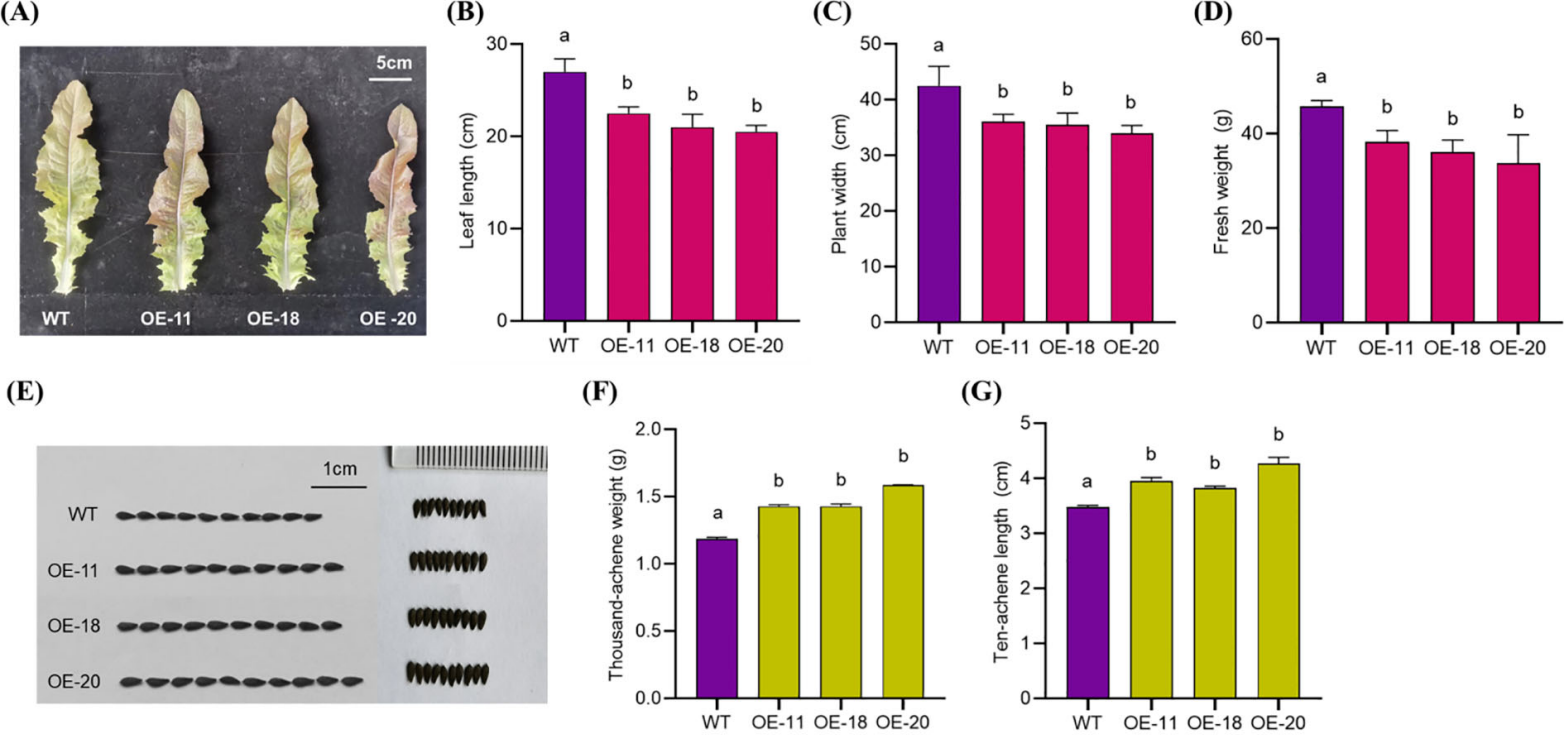
The phenotype of different overexpression *LsNAC46* transgenic lines and WT plants. **(A)** Representative leaf morphology of 45-day-old wild-type (WT, **‘**WD40AA**’** background) and three independent *LsNAC46*-overexpressing lines. **(B)** Leaf length and **(C)** plant width in WT and overexpression *LsNAC46* lines. **(D)** Fresh biomass weight in transgenic lines and WT (n = 4 biological replicates; 12 plants per replicate). **(E)** Achene morphology (scale bar: 1 cm) and **(F)** thousand-achene weight (TAW) in *LsNAC46*-OE lines and WT. **(G)** The length of ten achenes in OE lines and WT (thousand-achene weight and ten achene length; n = 3 per line). Values are means ± SD. Letters indicate significant differences between treatments based on Tukey’s test (P< 0.05).

A comprehensive evaluation of agronomic traits of 45-day-old plants revealed that OE-11, OE-18, and OE-20 had significantly lower plant width, leaf length, and fresh weight values per plant compared with WT ‘WD40AA’ (**Figures 4A–D**). However, after achene harvesting, the achene size, thousand achene weight, and achene length and width of overexpressing plants were significantly higher than WT plants (**Figures 4E–G**).

### 3.5 Overexpression of *LsNAC46* reduces heat tolerance in lettuce

**Figure 5:**
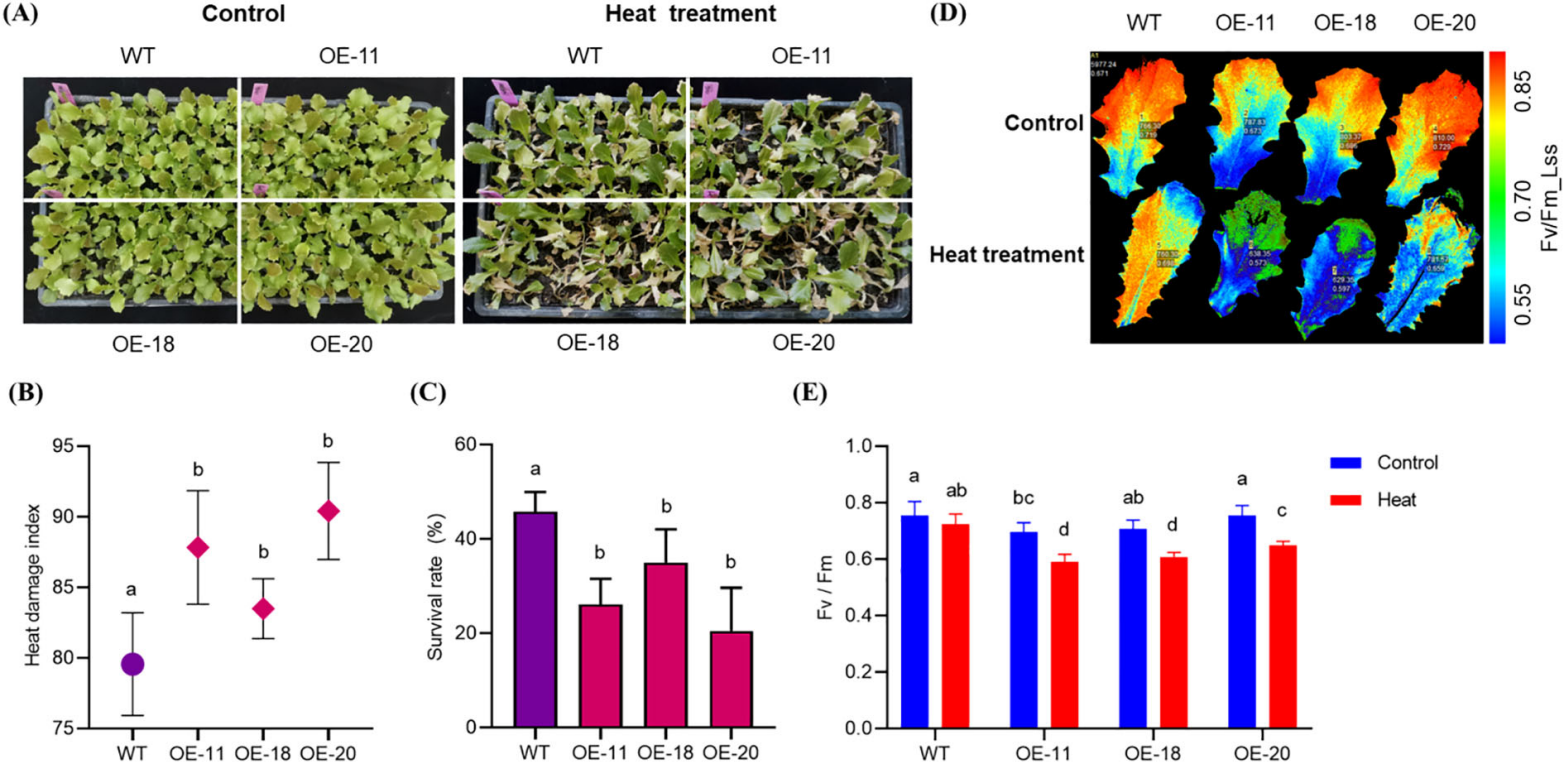
The phenotype of different overexpression LsNAC46 transgenic lines and WT plants under heat stress(The 25-day-old seedlings). **(A)** Comparative phenotypes of wild-type (WT, ‘WD40AA’ background) and three independent LsNAC46-OE lines (OE-11, OE-18, OE-20) under 72 h heat stress (16 h light/38°C, 8 h dark/33°C). **(B)** Heat damage index of WT and overexpression lettuce lines after 72 h treatment. **(C)** Survival rates post-recovery of WT and overexpression lettuce lines under normal conditions (16 h light/24°C, 8 h dark/20°C) for 48 h. **(D)** The images of PSII maximum photochemical efficiency (Fv/Fm) in WT and overexpression lettuce lines captured after 20 min dark adaptation. **(E)** Quantification of Fv/Fm values in WT and overexpression lettuce lines leaves, where Fm denotes maximal fluorescence and Fv represents variable fluorescence. Data are presented as mean ± SD (n = 3 biological replicates; 24 seedlings per replicate). For the bar graphs, quantities with the same lowercase letters above the bar denote quantities where statistical differences could not be detected by one-way ANOVA followed by Tukey’s multiple comparisons test (p< 0.05), while quantities with different lowercase letters denote groups with statistically significant differences.

After heat stress treatment of 25-day-old plants for 3 days, the overexpressing lines OE-11, OE-18 and OE-20 were more susceptible to heat stress than the WT plants (**Figure 5A**) with more weak plant and yellow leaves. The heat damage index of the WT plants was 80 compared to 88, 84 and 91 for the OE-11, OE-18, and OE-20 lines, respectively (**Figure 5B**). After 2 days of recovery at normal growth temperature after heat stress, the survival rates of OE-11, OE-18, and OE-20 overexpressing plants (26%, 35%, and 21%, respectively) were significantly lower than that of WT (46%) (**Figure 5C**). Chlorophyll fluorescence imaging further confirmed that the *LsNAC46*-overexpressing plants were more susceptible to heat damage, as they had significantly lower Fv/Fm values after being subjected to heat stress compared to the WT (decreased from 0.719 to 0.698): the Fv/Fm of OE-11 decreased from 0.697 to 0.592; OE-18, decreased from 0.708 to 0.608, and OE-20, decreased from 0.755 to 0.648 (**Figures 5D, E**). These results indicate that *LsNAC46* negatively regulates the heat stress response in lettuce.

### 3.6 Overexpression of *LsNAC46* significantly reduces the accumulation of TP, PRO, and PAL during heat stress

**Figure 6:**
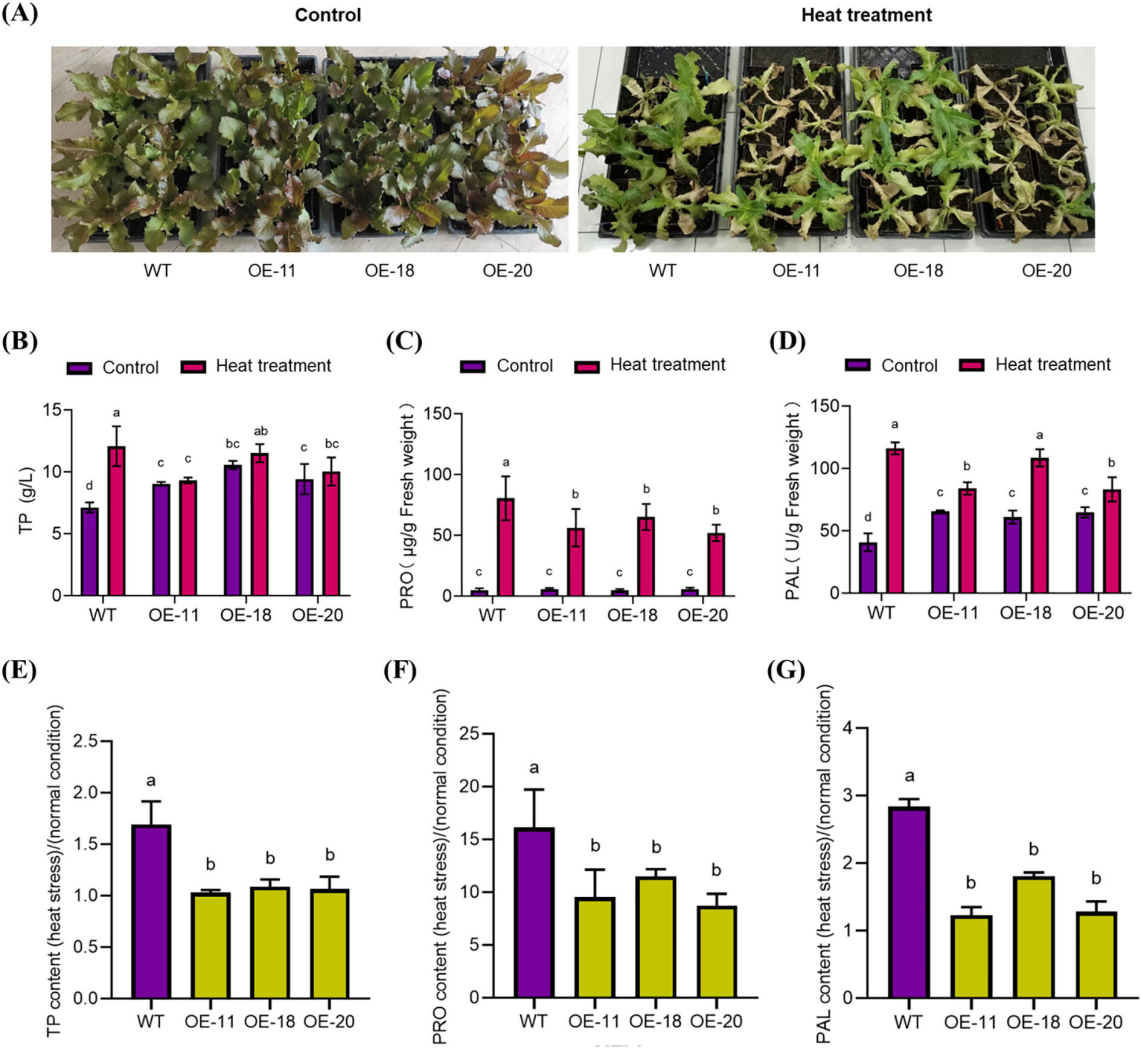
The phenotype and key physiological parameters of different overexpression *LsNAC46* transgenic lines and WT plants under heat stress (The 45-day-old seedlings). **(A)** Phenotypes of wild-type (WT, ‘WD40AA’ background) and three independent LsNAC46-overexpressing lines (OE-11, OE-18, OE-20) following 5-day heat stress (38°C/16 h light, 33°C/8 h dark). **(B)** Total phenolics (TP), **(C)** Proline (PRO), and **(D)** Phenylalanine ammo-nialyase (PAL) activity in leaves after heat stress. **(E-G)** Stress/normal condition accumulation ratios for **(E)** TP (TP~stress~/TP~control~), **(F)** PRO (PRO~stress~/PRO~control~), and **(G)** PAL (PAL~stress~/PAL~control~). Data represent mean ± SD (n = 3 biological replicates; 8 seedlings per replicate). Values are means ± SD. **(B-D)** For the bar graphs, quantities with the same lowercase letters above the bar denote quantities where statistical differences could not be detected by one-way ANOVA followed by Tukey’s multiple comparisons test (p< 0.05), while quantities with different lowercase letters denote groups with statistically significant differences.

After 5 days of heat stress treatment at 45-day-old, the *LsNAC46*-overexpressing lines OE-11, OE-18, and OE-20 were more sensitive to heat stress and suffered greater heat damage compared with WT plants (**Figure 6A**).

The majority of soluble proteins in plants are enzymes involved in various metabolic pathways, which are an important physiological indicator. Measuring that content is an important for understanding the total metabolic level in plants and studying enzyme activity. Exposure to heat stress for 5 days increased the TP content in the leaves of WT lettuce but did not significantly change the TP content of the OE-11, OE-18, and OE-20 lines (**Figure 6B**). In order to more intuitively reflect the upward trend of TP content, the TP content after heat stress was compared with the TP content without heat stress. The results showed that the TP accumulation multiple of transgenic plants significantly decreased in comparison with WT (**Figure 6C**).

The multifunctional amino acid PRO can alleviate the damage caused by stress by stimulating protective mechanisms in plants; PRO also plays an important role in regulating osmotic pressure. After heat stress treatment, the PRO content significantly increased (**Figure 6D**) in all plants; however, PRO accumulation was significantly lower in the *LsNAC46*-overexpressing lines OE-11, OE-18, and OE-20 compared with the WT (**Figure 6E**).

PAL is a key enzyme in plant phenylpropanoid metabolism and is closely related to the synthesis of important secondary metabolites such as phytoalexins and lignin and is evaluated as an indicator of plant physiology and stress resistance. After heat stress treatment, PAL enzyme activity significantly increased (**Figure 6F**). However, the increase in PAL enzyme activity was significantly lower in the *LsNAC46*-overexpressing lines OE-11, OE-18, and OE-20 compared with the WT (**Figure 6G**).

## 4 Discussion

In numerous previous studies, it has been demonstrated that the NAC gene has a wide range of biological functions in crops such as rice and corn. In this study, we explored the function of *LsNAC46*, a NAC gene. *LsNAC46* was over-expressed in lettuce ‘WD40AA’. Multiple alignment of LsNAC46 and its homologous proteins in *Arabidopsis thaliana*, *Helianthus annuus*, and *Oryza sativa* revealed their NAC domains are similar. Furthermore, LsNAC46 has a NAC conserved domain in its N-terminus, and the LsNAC46 promoter region contains cis-acting elements related to stress and hormones (**Figure 1B**). This combinatorial presence of phytohormone-related and stress-inducible cis-elements suggests that NAC expression in lettuce may be coordinately regulated by abscisic acid signaling, ethylene responses, and MYB-mediated transcriptional networks under abiotic stresses, potentially explaining its involvement in complex stress adaptation mechanisms.

Whole-genome expression analysis showed most NAC genes are induced by at least one abiotic stress in *Arabidopsis* and rice (Fujita et al., 2004; Fang et al., 2015). Recent expression analysis of global NAC genes demonstrated that the majority of NAC genes in *Arabidopsis* are responsive to extreme temperatures (Shan et al., 2007). Our laboratory previously identified LsNAC46 as a transcription factor that was differentially expressed after heat treatment in lettuce (Xi et al., 2022). In this study, we observed the relative expression level of *LsNAC46* was higher in achene than in any other tissues (**Figure 2**). Overexpression of *LsNAC46* significantly enhanced lettuce seed dimensions (length: 15.6% increase) and thousand-seed weight (TSW: 24.9% increase vs. wild type) (**Figure 4**). Phylogenetic conservation of NAC transcription factors across angiosperms suggests potential utility of LsNAC46 homologs for yield improvement in cereal crops such as rice, similarly with *OsNAC10* (Jeong et al., 2010).

Current research on NAC-mediated tolerance to heat stress regulation has predominantly focused on NAC-mediated transcriptional activation of antioxidant biosynthesis pathways for reducing heat injury, including proline (Pro), total phenolics (TP) and phenylalanine ammonia-lyase (PAL). Proline can increase the cellular osmotic potential and protect the stability of biological membranes, and thus alleviate osmotic damage and oxidative damage (Neelam and Preeti, 2009). Overexpression of *ZmNAC07* regulated the accumulation of various stress metabolites, including PRO, and significantly enhanced heat stress tolerance in *Arabidopsis* (Xi et al., 2022). Kolupaev found PAL activity regulates flavonoid accumulation in wheat plants under heat stress treatment, and thereby improved the resistance of wheat seedlings to low-temperature stress (Kolupaev et al., 2018). Li found that under drought stress, the activity of PAL in the leaves and ears of wheat during the filling stage initially increased and then decreased, indicating that drought stress increased the activities of these enzymes (Li et al., 2020). Numerous studies have focused on the enhanced tolerance to heat stress conferred by overexpression NAC transcription factor in plants (Xi et al., 2022), However, several studies have reported that NAC (NAM/ATAF/CUC) transcription factors exhibit upregulated expression under heat stress conditions, while their overexpression paradoxically results in decreased thermotolerance in plants. For instance, where the *ataf1–2* and *ataf1–4* mutants had a significantly higher survival rate and fresh mass compared with WT plants, while the ATAF1 was induced by heat stress and overexpressing *ATAF1* plants exhibited a severely reduced tolerant to heat stress (Alshareef et al., 2022). Expression of NAC gene *SlJA2* was induced by heat stress (42 °C) and overexpression of the *SlJA2* leads to the opening of stomatal apertures, increased water loss, restricted proline synthesis, and decreased heat resistance in tobacco plants (Liu et al., 2017). ONAC095 is transcriptionally activated by drought stress; however, its induction paradoxically compromises drought tolerance in rice (*Oryza sativa*) (Huang et al., 2016). Our study show that *LsNAC46* was induced by heat stress (**Figure 3C**). However, the over expression of *LsNAC46* will reduce the heat tolerance under heat stress with higher heat damage index (**Figure 5B**). So the *LsNAC46* may have the summarily function with *ATAF1*, *SlJA2* in regulating heat stress or *OsNAC95* in regulating drought stress. In this study, the content of PRO in our study also increased under heat stress in WT and overexpressed lines plants (**Figure 6**), but the stress/normal condition accumulation ratios for PRO in over expression lines were lower than in WT. Similarly, the stress/normal condition accumulation ratios for PAL and TP content was also significantly lower in *LsNAC46*-overexpressing plants (**Figure 6**). So we hypothesize that over expression *LsNAC46* regulate lettuce tolerance to heat stress by suppressing the accumulation of key antioxidants, including proline, phenylalanine ammonia-lyase, and phenolic compound. These lower antioxidants likely disrupts reactive oxygen species (ROS) scavenging capacity, leading to excessive hydroxyl radical (•OH) and superoxide anion (O_2_^-^) accumulation under heat stress (38°C/16 h light, 33°C/8 h dark) in transgenic lines compared to wild-type controls.

This study conducted a preliminary exploration of the regulatory mechanisms in lettuce phenotype and heat tolerance. However, further improvement is needed for the functional complementation experiments, such as the supplementation of overexpression and knockout experiments for the same gene and receptor. Additionally, further analysis is required for the regulatory mechanisms of NAC upstream and downstream genes. But the over expression lines can give a preliminary exploration of the regulatory mechanisms in other species, such as *ZmNAC074* (Xi et al., 2022). What’s more, the functional redundancy of NAC genes had been reported (Javier and Angel, 2024), maybe the function will be supplement by other *LsNACs* when the *LsNAC46* were knock out.

In conclusion, we cloned the *LsNAC46* gene based on our previous study. It found that LsNAC46 has transcriptional activation activity and is expressed at the highest levels during achene development. We demonstrated that over-expression *LsNAC46* increased the seed weight under normal condition, and the higher heat damage index and decreased survival rate under heat stress in lettuce by lowering the accumulation of TP, PRO, PAL and Fv/Fm; and over expression *LsNAC46* lines can decreased the lettuce fresh weight. Hence, *LsNAC46* may play important roles in heat tolerance, growth and seed development. These physiological alterations resulted in elevated heat injury indices and diminished survival rates under thermal stress. This investigation may provide novel insights into the molecular mechanisms underlying lettuce’s response to heat stress, particularly highlighting the paradoxical role of NAC overexpression in exacerbating heat stress susceptibility by lower stress-related metabolites.

## Data Availability

The original contributions presented in the study are included in the article/**Supplementary Material**. Further inquiries can be directed to the corresponding author.

## Supplementary material

Supplementary Table 1 Analysis of cis reactive elements in the *LsNAC46* gene promoter of lettuce.

Supplementary Table 2 Sequences of primers used in this manuscript.

## References

[B1] Alshareef N. Otterbach S. Allu A. Woo Y. de Werk T. Kamranfar I.. (2022). NAC transcription factors ATAF1 and ANAC055 affect the heat stress response in Arabidopsis. Sci. Rep. 12, 11264. doi: 10.1038/S41598-022-14429-X, PMID: 35787631 PMC9253118

[B2] Choudhury F. Rivero R. Blumwald E. Mittler R. (2017). Reactive oxygen species, abiotic stress and stress combination. Plant J. 90, 856–867. doi: 10.1111/tpj.13299, PMID: 27801967

[B3] Du L. Yang X. Xu A. Wei H. Yang X. Wei S.. (2021). Identification of NAC gene family in leaf lettuce and their expression under heat stress, (in Chinese). Mol. Plant Breed. 19, 6265–6276. doi: 10.13271/j.mpb.019.006265

[B4] Fang Y. Liao K. Du H. Xu Y. Song H. Li X.. (2015). A stress-responsive NAC transcription factor SNAC3 confers heat and drought tolerance through modulation of reactive oxygen species in rice. J. Exp. Bot. 66, 6803–6817. doi: 10.1093/jxb/erv386, PMID: 26261267 PMC4623689

[B5] Fujita M. Fujita Y. Maruyama K. Seki M. Hiratsu K. Ohme-Takagi M.. (2004). A dehydration-induced NAC protein, RD26, is involved in a novel ABA-dependent stress-signaling pathway. Plant J. 39, 863–876. doi: 10.1111/j.1365-313X.2004.02171.x, PMID: 15341629

[B6] Guan C. Wu B. Ma S. Zhang J. Liu X. Wang H.. (2023). Genome-wide characterization of LBD transcription factors in switchgrass (Panicum virgatum L.) and the involvement of PvLBD12 in salt tolerance. Plant Cell Rep. 42, 735–748. doi: 10.1007/S00299-023-02989-9, PMID: 36806743

[B7] Guan Q. Yue X. Zeng H. Zhu J. (2014). The protein phosphatase RCF2 and its interacting partner NAC019 are critical for heat stress-responsive gene regulation and thermotolerance in Arabidopsis. Plant Cell. 26, 438–453. doi: 10.1105/tpc.113.118927, PMID: 24415771 PMC3963588

[B8] Guo W. Zhang J. Zhang N. Xin M. Peng H. Hu Z.. (2015). The wheat NAC transcription factor taNAC2L is regulated at the transcriptional and post-translational levels and promotes heat stress tolerance in transgenic Arabidopsis. PLoS One 10, e0135667. doi: 10.1371/journal.pone.0135667, PMID: 26305210 PMC4549282

[B9] Hare P. Cress W. Van Staden J. (2010). Dissecting the roles of osmolyte accumulation during stress. Plant Cell Environ. 21, 535–553. doi: 10.1046/j.1365-3040.1998.00309.x

[B10] Huang L. Hong Y. Zhang H. Li D. Song F. (2016). Rice NAC transcription factor ONAC095 plays opposite roles in drought and cold stress tolerance. BMC Plant Biol. 16, 203. doi: 10.1186/s12870-016-0897-y, PMID: 27646344 PMC5029094

[B11] Javier F. Angel J. (2024). Transcriptional control of seed life: new insights into the role of the NAC family. Int. J. Mol. Sci. 25, 41. doi: 10.3390/IJMS25105369, PMID: 38791407 PMC11121595

[B12] Jenni S. Truco M. Michelmore R. (2013). Quantitative trait loci associated with tipburn, heat stress-induced physiological disorders, and maturity traits in crisphead lettuce. Theor. Appl. Genet. 126, 3065-3079. doi: 10.1007/s00122-013-2193-7, PMID: 24078012

[B13] Jeong J. Kim Y. Baek K. Jung H. Ha S. Do Choi Y.. (2010). Root-specific expression of OsNAC10 improves drought tolerance and grain yield in rice under field drought conditions. Plant Physiol. 153, 185–197. doi: 10.1104/pp.110.154773, PMID: 20335401 PMC2862432

[B14] Kaliyugam S. Rinku S. Krishan K. Shiv K. Firoz H. Nepolean T. (2014). Genome-wide identification and expression pattern of drought-responsive members of the NAC family in maize. Meta Gene. 2, 2407–2417. doi: 10.1016/j.mgene.2014.05.001, PMID: 25606426 PMC4287890

[B15] Kolupaev Y. Horielova E. Yastreb T. Popov Y. Ryabchun N. (2018). Phenylalanine ammonia-lyase activity and content of flavonoid compounds in wheat seedlings at the action of hypothermia and hydrogen sulfide donor. Ukr. Biochem. J. 90, 12–20. doi: 10.15407/ubj90.06.012

[B16] Le D. Nishiyama R. Watanabe Y. Mochida K. Yamaguchi-Shinozaki K. Shinozaki K.. (2011). Genome-wide survey and expression analysis of the plant-specific NAC transcription factor family in soybean during development and dehydration stress. DNA Res. 18, 263–276. doi: 10.1093/dnares/dsr015, PMID: 21685489 PMC3158466

[B17] Li X. Zhang X. Liu G. Tang Y. Lv J. (2020). The spike plays important roles in the drought tolerance as compared to the flag leaf through the phenylpropanoid pathway in wheat. Plant Physiol. Biochem. 152, 100–111. doi: 10.1016/j.plaphy.2020.05.002, PMID: 32408177

[B18] Liu Z. Yue M. Yang D. Zhu S. Ma N. Meng Q. (2017). Over-expression of *SlJA2* decreased heat tolerance of transgenic tobacco plants via salicylic acid pathway. Plant Cell Rep. 36, 529–542. doi: 10.1007/s00299-017-2100-9, PMID: 28155114

[B19] Neelam M. Preeti S. (2009). Effect of salicylic acid on proline metabolism in lentil grown under salinity stress. Plant Sci. 177, 181–189. doi: 10.1016/j.plantsci.2009.05.007

[B20] Mohammed N. Ramaswamy M. Akhter M. Satoh K. Kondoh H. Ooka H.. (2010). Genome-wide analysis of NAC transcription factor family in rice. Gene 465, 30–44. doi: 10.1016/j.gene.2010.06.008, PMID: 20600702

[B21] Mohanta T. Yadav D. Khan A. Hashem A. Tabassum B. Khan A.. (2020). Genomics, molecular and evolutionary perspective of NAC transcription factors. PLoS One 15, e0231425. doi: 10.1371/journal.pone.0231425, PMID: 32275733 PMC7147800

[B22] Qu A. Ding Y. Jiang Q. Zhu C. (2013). Molecular mechanisms of the plant heat stress response. Biochem. Biophys. Res. Commun. 432, 203–207. doi: 10.1016/j.bbrc.2013.01.104, PMID: 23395681

[B23] Shah F. Huang J. Cui K. Nie L. Shah T. Chen C.. (2011). Impact of high-temperature stress on rice plant and its traits related to tolerance. J. Agric. Sci. 149, 545–556. doi: 10.1017/S0021859611000360

[B24] Shahnejat-Bushehri S. Mueller-Roeber B. Balazadeh S. (2012). Arabidopsis NAC transcription factor JUNGBRUNNEN1 affects thermomemory-associated genes and enhances heat stress tolerance in primed and unprimed conditions. Plant Signal. Behav. 7, 1518–1521. doi: 10.4161/psb.22092, PMID: 23073024 PMC3578882

[B25] Shan D. Huang J. Yang Y. Guo Y. Wu C. Yang G.. (2007). Cotton GhDREB1 increases plant tolerance to low temperature and is negatively regulated by gibberellic acid. New Phytol. 176, 70–81. doi: 10.1111/J.1469-8137.2007.02160.X, PMID: 17803642

[B26] Shang H. Li W. Zou C. Yuan Y. (2013). Analyses of the NAC transcription factor gene family in Gossypium raimondii Ulbr: chromosomal location, structure, phylogeny, and expression patterns. J. Integr. Plant Biol. 55, 663–676. doi: 10.1111/jipb.12085, PMID: 23756542

[B27] Sperotto R. Ricachenevsky F. Duarte G. Boff T. Lopes K. Sperb E.. (2009). Identification of up-regulated genes in flag leaves during rice grain filling and characterization of OsNAC5, a new ABA-dependent transcription factor. Planta 230, 985–1002. doi: 10.1007/s00425-009-1000-9, PMID: 19697058

[B28] Su W. Tao R. Liu W. Yu C. Yue Z. He S.. (2020). Characterization of four polymorphic genes controlling red leaf colour in lettuce that have undergone disruptive selection since domestication. Plant Biotechnol. J. 18, 479–490. doi: 10.1111/pbi.13213, PMID: 31325407 PMC6953203

[B29] Swati P. Pranav P. Prem S. Manoj P. (2012). NAC proteins: regulation and role in stress tolerance. Trends Plant Sci. 17, 369–381. doi: 10.1016/j.tplants.2012.02.004, PMID: 22445067

[B30] Wang Y. Sun S. Feng X. Li N. Song X. (2024). Two lncRNAs of Chinese cabbage confer Arabidopsis with heat and drought tolerance. Veg. Res. 4, e029. doi: 10.48130/vegres-0024-0029

[B31] Wei S. Zhang L. Huo G. Ge G. Luo L. Yang Q.. (2021). Comparative transcriptomics and metabolomics analyses provide insights into thermal resistance in lettuce (*Lactuca sativa* L.). Sci. Hortic. 289, 110423. doi: 10.1016/J.SCIENTA.2021.110423

[B32] Xi Y. Ling Q. Zhou Y. Liu X. Qian Y. (2022). ZmNAC074, a maize stress-responsive NAC transcription factor, confers heat stress tolerance in transgenic Arabidopsis. Front. Plant Sci. 13. doi: 10.3389/FPLS.2022.986628, PMID: 36247610 PMC9558894

[B33] Yamaguchi S. Shinozaki K. (2007). Gene networks involved in drought stress response and tolerance. J. Exp. Bot. 58, 221–227. doi: 10.1093/jxb/erl164, PMID: 17075077

